# Pressurized lacrimal duct irrigation for neonatal dacryocystitis: a pilot study of safety, effectiveness, and intervention timing

**DOI:** 10.3389/fmed.2026.1969796

**Published:** 2026-10-28

**Authors:** Xiaofang Zhan, Han Wu, Mani Vimalin Jeyalatha, Lingyu Zhang, Sijie Lin, Yiming Wu, Shangkun Ou

**Affiliations:** 1 Xiamen Eye Center and Eye Institute of Xiamen University, School of Medicine, Xiamen, Fujian, China; 2 Fujian Key Laboratory of Corneal & Ocular Surface Diseases, Xiamen Key Laboratory of Corneal & Ocular Surface Diseases, Xiamen, Fujian, China; 3 Translational Medicine Institute, Xiamen Eye Center, Xiamen University, Xiamen, Fujian, China; 4 Fujian Provincial Key Laboratory of Ophthalmology and Visual Science, Fujian Engineering and Research Center of Eye Regenerative Medicine, School of Medicine, Xiamen University, Xiamen, Fujian, China; 5 Tianjin Key Laboratory of Retinal Functions and Diseases, Tianjin Branch of National Clinical Research Center for Ocular Disease, Eye Institute and School of Optometry, Tianjin Medical University Eye Hospital, Tianjin, China; 6 Department of Biomedical Sciences, School of Infection, Inflammation and Immunology, College of Medicine and Health, University of Birmingham, Birmingham, United Kingdom; 7 Department of Ophthalmology, The Affiliated Hospital of Guizhou Medical University, Guiyang, Guizhou, China

**Keywords:** neonatal dacryocystitis, pressurized lacrimal duct irrigation, nasolacrimal duct obstruction, pediatric ophthalmology, modified irrigation needle

## Abstract

**Objective:**

The study aims to assess the effectiveness, safety, and age-related outcomes of a pressurized lacrimal duct irrigation technique in neonatal dacryocystitis.

**Methods:**

The study analyzed the outcomes of 145 infants (166 eyes) diagnosed with neonatal dacryocystitis at Affiliated Xiamen Eye Center of Xiamen University. Participants were categorized into three age-based cohorts: Group A (3–5 months), Group B (6–8 months), and Group C (9–11 months). A modified pressurized lacrimal duct irrigation technique was performed to unblock the lacrimal duct. The lacrimal duct patency and incidence of related complications were observed and recorded after the procedure. The infants were followed up for a week, and the procedure was repeated if the blockage persisted.

**Results:**

The overall patency rate was 83.73%, with rates of 94.83% in Group A, 84.48% in Group B, and 70.00% in Group C. Specifically, patency was achieved after the first intervention in 61 eyes (36.75%), after a second intervention in 51 eyes (30.72%), and after a third intervention in 27 eyes (16.27%). Complications such as lacrimal punctum tear, punctal ectropion, conjunctival hemorrhage, and corneal epithelial exfoliation were not observed, but four eyes presented with lacrimal duct mucosal hemorrhage and one eye had a pseudo-tract post-flushing.

**Conclusions:**

The improved pressurized lacrimal duct irrigation technique for neonatal dacryocystitis was found to be safe and effective, with higher patency rates observed in infants aged 3–5 months.

## 1 Introduction

Dacryocystitis is among the most common ophthalmic diseases in children, particularly neonates (1, 2). Neonatal dacryocystitis is commonly secondary to congenital nasolacrimal duct obstruction (CNLDO). Epidemiological data indicate that CNLDO affects 5%–20% of full-term newborns (3, 4). This condition primarily arises from the non-perforation of Hasner's valve at the distal end of the nasolacrimal duct during development or from obstruction caused by epithelial debris blocking the duct (5–7). Such obstruction impedes tear drainage, leading to tear stasis and bacterial accumulation in the lacrimal sac. This accumulation predisposes the infant to secondary infection and ultimately triggers neonatal dacryocystitis (8).

Clinically, affected infants present with excessive tearing accompanied by viscous or purulent discharge; some also exhibit medial canthal erythema or eczema. Compression of the lacrimal sac or lacrimal irrigation typically yields purulent reflux through the punctum. Without prompt intervention, severe complications may develop, including keratitis, acute dacryocystitis, lacrimal sac abscess, and other serious consequences (9, 10). Severe acute dacryocystitis can lead to systemic infection and intracranial infection, endangering the life of the infant (11).

Currently, the primary treatment for neonatal dacryocystitis involves lacrimal sac massage combined with topical antibiotic therapy (12, 13). When conservative treatment fails, lacrimal duct irrigation or surgical interventions, such as lacrimal duct probing, lacrimal intubation, and dacryocystorhinostomy (DCR), should be considered (14–19). Compared to surgical options, lacrimal duct irrigation offers greater procedural convenience and significantly lower invasiveness in infants, making it the preferred approach for refractory cases (20). However, clinical studies indicate that the patency rate and clinical outcomes of irrigation depend on infant age, and the optimal treatment window after birth remains debated.

In clinical practice, we observed that pressurized lacrimal duct irrigation demonstrated a high patency rate in managing neonatal dacryocystitis. Based on this finding, we designed a prospective interventional study to evaluate the safety and efficacy of this modified technique across different infant age groups.

## 2 Methods

### 2.1 Study design

A prospective interventional study was designed, and infants presenting with neonatal dacryocystitis at the outpatient clinic of Affiliated Xiamen Eye Center of Xiamen University from 1 June 2020 to 31 May 2022 were enrolled. The study was approved by the ethics committee of the Affiliated Xiamen Eye Center of Xiamen University and performed in accordance with the tenets of the Declaration of Helsinki.

### 2.2 Patient population

The study involved a total of 145 infants (166 eyes) diagnosed with neonatal dacryocystitis at the Affiliated Xiamen Eye Center of Xiamen University. The cohort was divided into three groups based on age: Group A (3–5 months), Group B (6–8 months), and Group C (9–11 months). As an exploratory pilot study, a formal pre-study sample size calculation was not performed; instead, consecutive eligible infants were enrolled during the study period to provide preliminary safety and efficacy data.

Clinical characteristics and demographic details were recorded. Patients were diagnosed with neonatal dacryocystitis based on their history, signs, and symptoms. The infants exhibited excessive tearing, mucopurulent discharge from the lacrimal punctum upon lacrimal sac squeezing, and poor response to 2 weeks of conservative treatment with antibiotic eye drops (four times daily) and lacrimal sac massage (two times daily), or recurrence after discontinuation of treatment. Infants presenting with the following symptoms were excluded from the study: congenital punctal atresia; excessive tearing caused by conjunctivitis; excessive tearing caused by entropion and trichiasis; acute inflammatory conditions such as acute dacryocystitis; and surgical contraindications including upper respiratory tract infection, fever, diarrhea, coagulopathy, and severe facial developmental malformations. In addition, patients younger than 3 months or older than 11 months were also excluded. We provided health guidance to the child's guardians, addressed their concerns, and gained their trust. Meanwhile, we also explained the purpose, procedure, possible complications, and corresponding treatment methods during the lacrimal duct irrigation to obtain their support and cooperation. Written informed consent was obtained from all guardians of participants.

### 2.3 Pressurized lacrimal duct irrigation

Prior to irrigation procedures, a modified steel irrigation needle was prepared (Figure 1). We modified a #6 stainless steel irrigation needle; the tip was polished into a blunt bevel by the same engineer according to the standardized protocol described in our patent (21), ensuring that the diameter of the modified irrigation needle tip was 0.5 mm. The blunt tip of the modified irrigation needle has a smaller diameter, which facilitates smooth insertion into the narrow lacrimal duct of infants, while the wider needle body allows for increased irrigation pressure and prevents fluid reflux (Figure 1C). In addition, the following equipment was prepared: a 2-mL disposable syringe, cotton swabs, cotton balls, a punctum dilator, topical anesthetic, and sterile saline solution.

**Figure 1:**
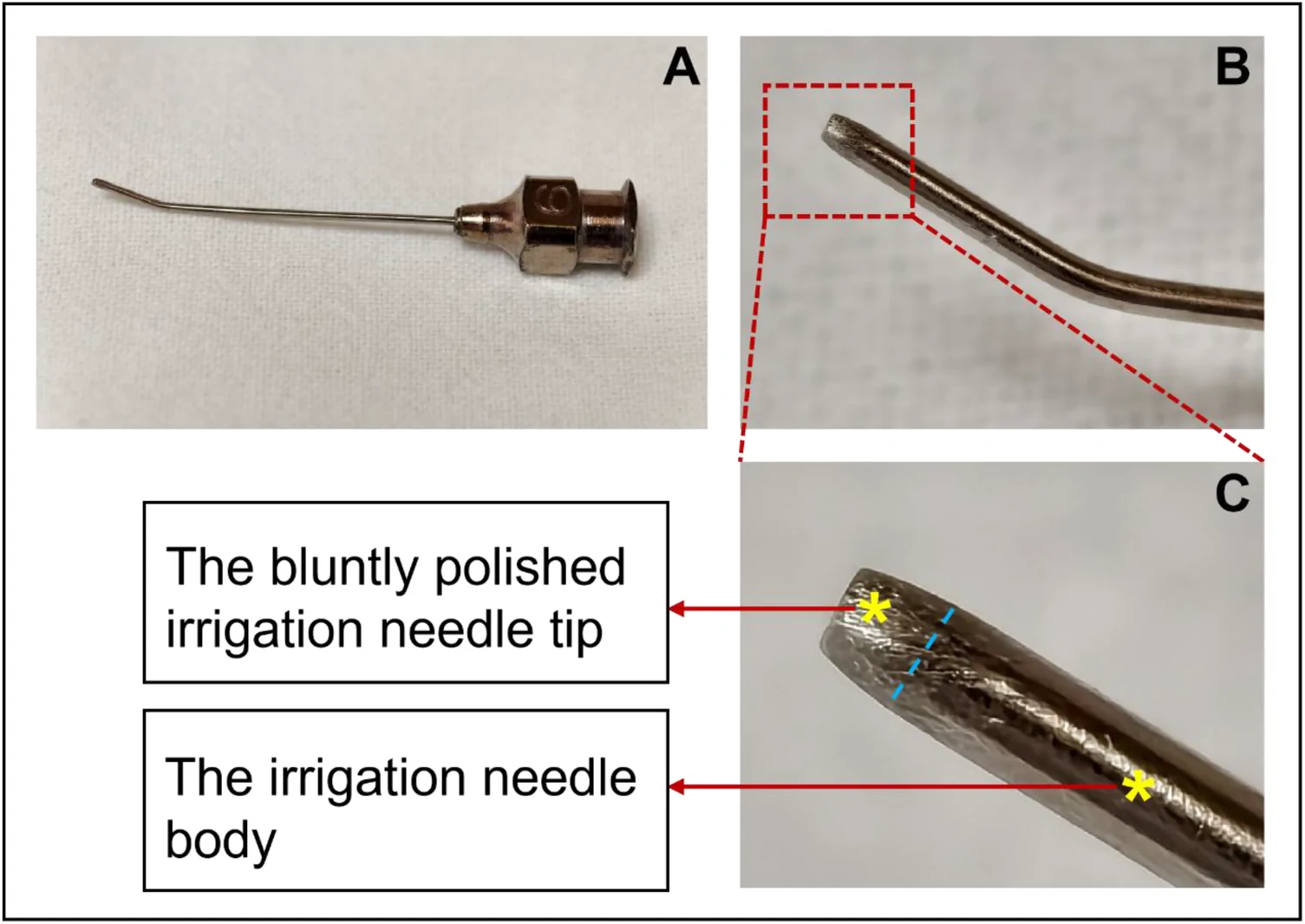
The modified stainless steel irrigation needle. **(A)** The modified #6 stainless steel irrigation needle. **(B)** The tip and body of the modified irrigation needle. **(C)** Magnified view of the bluntly polished tip and the wider body of the modified irrigation needle.

After three drops of proparacaine were instilled into the conjunctival sac, the infant was placed in the supine position, and the adult assisted in applying pressure to immobilize the infant's head and body without pressing on the infant's fontanelle (Figure 2A). The extraocular discharge was removed with sterile saline swabs, the lower eyelid was pulled down to fully expose the lower lacrimal punctum, and it was dilated with a dilator (Figure 2B). A preloaded 2 mL saline irrigator was vertically inserted into the lower lacrimal punctum to a depth of 1.5 mm (Figure 2C). After insertion, the needle was turned horizontally and passed into the lacrimal duct until it touched the bone wall. With the needle fixed in position, 0.5 mL of saline was slowly instilled (Figure 2D). All the above operations were carried out by the same surgeon. Simultaneously, fluid reflux and its characteristics were observed. The upper eyelid was lifted to expose the upper lacrimal punctum and pressed against the bone wall with a cotton swab by the same surgical assistant. Then, irrigated was performed while observing the fluid flow, bleeding, and false tract. If the infant swallowed or fluid flowed through the nostril, it indicated an unblocked lacrimal duct. If the initial irrigation was unsuccessful, the procedure was repeated every other week for three consecutive sessions, and any smooth irrigation was considered successful. If the infant exhibited cough and cyanosis, nasal bleeding, or a swollen eyelid, the irrigation was immediately terminated, and the status of the infant was assessed. The short-term outcomes included no epiphora, normalized tear flow, and ocular secretion; in addition, there was no reobstruction during follow-up. After successful irrigation, the infants were followed up at the outpatient clinic 2 weeks postoperatively and subsequently by telephone at 1, 3, and 6 months.

**Figure 2:**
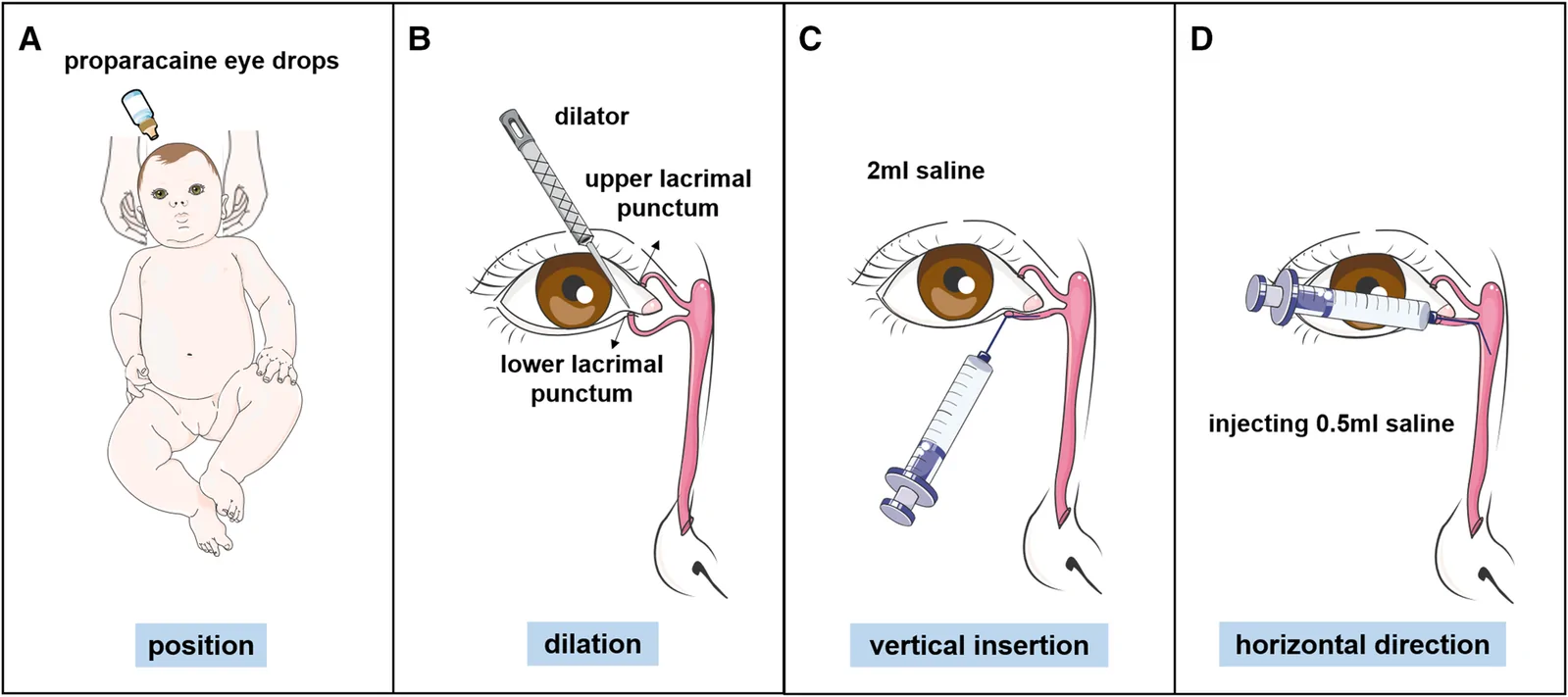
The specific operation of pressurized lacrimal duct irrigation. **(A)** Position and anesthesia for pressurized lacrimal duct irrigation in infants. **(B)** Expose and dilate the lower lacrimal punctum. **(C)** Insert a 2 mL saline irrigator fitted with the modified irrigation needle vertically into the lower lacrimal punctum. **(D)** Turn the irrigation needle horizontally, pass it into the lacrimal duct and inject 0.5 mL of saline.

### 2.4 Statistical analyses

All statistical analyses were performed using SPSS software (version 26.0; IBM Corp., Armonk, NY, USA). Continuous data were expressed as mean ± standard deviation (SD); comparisons between two groups were analyzed using Student's *t*-test, while comparisons among multiple groups were analyzed using one-way ANOVA. Categorical data were expressed as counts and percentages (%) and analyzed using the chi-square (*χ*^2^) test. A value of *P* < 0.05 was considered statistically significant.

## 3 Results

### 3.1 Demographic details

A total of 145 cases (166 eyes) of neonatal dacryocystitis were admitted during the 2 years. The demographic details of the study population are shown in Table 1, including 59 males and 86 females, with 124 unilateral and 21 bilateral dacryocystitis. The patients were aged 3–11 months, with a mean age of 7.01 ± 2.55 months. In group A, there were 50 infants (58 eyes), with a mean age of 4.18 ± 0.83 months. In group B, there were 50 infants (58 eyes), with a mean age of 7.00 ± 0.83 months. In group C, there were 45 infants (50 eyes), with a mean age of 10.11 ± 0.85 months. The difference in mean age between the three groups was statistically significant (*P* < 0.05).

**Table 1:** Group of patients and their general information.

Group		A	B	C	*P*
Mean age (months)		4.18 ± 0.83	7.00 ± 0.83	10.11 ± 0.85	<0.05
Gender	Male	18	22	17	0.693
	Female	32	28	28	
Mode of delivery	Eutocia	42	44	41	0.573
	C-section	8	6	4	
Mean gestational age (weeks)		37.5 ± 2.1	38.1 ± 2.5	38.2 ± 1.7	0.2182

### 3.2 The effectiveness and safety of the improved pressurized lacrimal duct irrigation for infants with dacryocystitis

Nasolacrimal duct flushing achieved overall patency in 139 of the 166 eyes (83.73%). Specifically, patency was achieved after the first intervention in 61 eyes (36.75%), after a second intervention in 51 eyes (30.72%), and after a third intervention in 27 eyes (16.27%). The overall patency rate was 94.83% in group A, 84.48% in group B, and 70.00% in group C. As shown in Table 2, the patency rates of the three groups were statistically significant (*χ*^2^ = 42.595, *P* < 0.001), with younger infants requiring significantly fewer irrigation procedures to achieve patency compared to older cohorts.

**Table 2:** Effect of the performance of pressure lacrimal irrigation for infants with dacryocystitis in different age groups (eye).

Group	Smooth (flush times)			Obstructed	Total	*χ* ^2^	*P*
	1	2	3				
A	36	15	4	3	58	42.595	<0.001
B	19	23	7	9	58		
C	6	13	16	15	50		

In our study, five eyes developed complications during irrigation, including lacrimal duct mucosal hemorrhage in four eyes and a false tract in one eye. No other complications occurred, such as lacrimal punctum tears, ectropion, conjunctival hemorrhage, or corneal epithelial detachment. When hemorrhage or a false tract occurred, irrigation was stopped, and the nasal bridge was immediately pressed with a cotton ball for 5–10 min. For the four cases of lacrimal duct mucosal hemorrhage, pressurized irrigation was reapplied at the 1-week follow-up without obstruction or further bleeding. The overall complication rate was 3.01% in our improved pressurized lacrimal duct irrigation for neonatal dacryocystitis. As Table 3 shows, the complication rate in group A was 1.72%, lower than in group B (3.45%) and group C (4%). This explains the finding that younger infants had fewer complications after pressurized lacrimal duct irrigation. However, due to the small sample size of complications, there was no statistical significance between the three groups (*P* > 0.05).

**Table 3:** Complications post intervention.

Group	A	B	C	*P*
Complication	1	2	2	0.766
Complication rate (%)	1.72	3.45	4	

As shown in Table 2, a total of 139 eyes achieved patency through the pressurized lacrimal duct irrigation in our study. To evaluate the postoperative efficacy and long-term reobstruction rates, the infants were followed up at the outpatient clinic at 2 weeks postoperatively and subsequently by telephone at 1, 3, and 6 months. All subjects completed both the short-term follow-up and the long-term telephone follow-up. At the 2-week postoperative outpatient follow-up, all guardians reported no recurrence of symptoms such as excessive tearing accompanied by viscous or purulent discharge, and slit-lamp examination revealed no signs associated with dacryocystitis. In addition, evaluative lacrimal duct irrigation was performed using a #5 ordinary irrigation needle, confirming that the lacrimal ducts were patent in all infants. In addition, during the telephone follow-ups at 1, 3, and 6 months postoperatively, all guardians reported no recurrence of dacryocystitis-related symptoms, such as excessive tearing accompanied by viscous or purulent discharge. The results demonstrated that no recurrence of obstruction was observed at 2 weeks after successful pressurized lacrimal duct irrigation in any infants across groups A, B, and C, and no recurrence of dacryocystitis-related symptoms was observed at 6 months after irrigation.

## 4 Discussions

The etiology of infantile dacryocystitis is mainly due to the incomplete degeneration of the embryonic Hasner's valve at the distal end of the nasolacrimal duct, resulting in the accumulation of tears in the lacrimal sac, which eventually harbors bacteria and leads to purulent discharge and lacrimation in children (22–25). The relief of CNLDO constitutes a pivotal step in management. An authoritative expert consensus states that for CNLDO in infants, a stepwise therapeutic strategy should be followed, progressing from conservative to surgical management, from simple to complex interventions, from non-invasive to minimally invasive procedures, and from single to combined modalities (26). Accordingly, in clinical practice, irrigation is frequently regarded as the preferred approach for removing obstruction following the failure of antibiotic therapy and lacrimal sac massage (22).

Unlike conventional irrigation, pressurized lacrimal irrigation reopens the obstructed nasolacrimal duct by increasing fluid pressure during the procedure. Our previous study, conducted before the improvement of the irrigation needle, showed a patency rate of 72.7% with ordinary pressurized irrigation and 5.5% with conventional irrigation in neonatal dacryocystitis, indicating that appropriate pressure can significantly improve the patency rate of neonatal dacryocystitis (27).

Pressurized lacrimal irrigation in infants is technically challenging, and operator skill directly influences procedural effectiveness. Infants are prone to false tracts and bleeding during irrigation because of their delicate mucosa, which increases procedural discomfort. To improve the patency rate of pressurized irrigation, we first occluded the upper lacrimal punctum with a cotton swab during the pressurized irrigation of the lower lacrimal punctum, thereby increasing the hydraulic pressure to break through the residual membrane.

In this study, we modified the lacrimal irrigation needle with a smaller tip and a thicker body. The smaller tip facilitated needle insertion during lacrimal irrigation, thereby avoiding damage to the lacrimal punctum due to overdistention, while the thicker body prevented reflux of irrigation fluid from the lower lacrimal duct, thereby increasing the irrigation pressure exerted on Hasner’s valve and improving the patency rate. We achieved an overall patency rate of 83.73% with the modified irrigation needle combined with pressure on the superior lacrimal duct. In a related report, only 97 of 412 eyes (386 outpatient cases) were cured by pressurized lacrimal duct irrigation without pressure on the superior lacrimal punctum, yielding a patency rate of only 31.1% (28). The optimal timing of lacrimal irrigation in infants after birth remains debated. For infants younger than 3 months, pressurized lacrimal irrigation is not recommended because the lacrimal puncta and canaliculi are relatively small, making localization for irrigation challenging and increasing the risk of damage to these structures (29). Moreover, because their nervous systems are not fully mature, infants are prone to choking during irrigation, which may lead to aspiration, asphyxia, and other risks. Therefore, massage combined with antibiotic treatment is generally used as conservative treatment in infants aged 1–3 months (18, 22, 24). If this approach proves ineffective, irrigation and nasolacrimal probing are performed once the child is at least 3 months old. To date, reliable studies defining the optimal timing of lacrimal irrigation for neonatal dacryocystitis are still lacking.

Our study analyzed the safety and efficacy of improved pressurized lacrimal duct irrigation in infants with dacryocystitis at different ages. The study showed that the overall patency rate of pressure irrigation was 94.83% in group A, 84.48% in group B, and 70.00% in group C, indicating that the younger the infant, the higher the patency rate. As shown in Table 2, in the 58 eyes of group A, the highest patency rate was achieved with the first irrigation (36/58, 62.07%); in the 58 eyes of group B, the highest patency rate was achieved with the second irrigation (23/58, 39.66%); and in the 50 eyes of group C, the highest patency rate was achieved with the third irrigation (16/50, 32.00%). Clearly, the younger the infant, the fewer irrigations were required to achieve patency. This is likely due to the shorter duration of the disease in younger infants and the thinner membrane at the bottom of the nasolacrimal canal, which is more susceptible to opening under the effect of irrigation. In contrast, in older infants with dacryocystitis, as they grow older, chronic inflammation repeatedly irritates the lacrimal sac and nasolacrimal duct over a long period, which may lead to thick and hard membranous tissue and increasingly obstructive residual membranes. At the same time, the scar formed by inflammatory irritation further aggravates the narrowing of the lacrimal duct, increasing the difficulty of treatment and the number of irrigations required.

Regardless of the age of the infants, lacrimal duct mucosal thickening was observed. Long-term inflammation of the lacrimal sac results in cystic cavity inflammation and fibrous scarring of the lacrimal sac, altering the anatomy. Thus, when the probe is forced repeatedly, the risk of complications like hemorrhage increases. The occurrence of a false tract in one case may be related to the misalignment of the infant's head not being secured or improper manipulation. The infant did not swallow, and there was no nasal reflux when the resistance suddenly decreased during irrigation; irrigation should be stopped immediately, and pressure should be applied for 5 min. The pressurized lacrimal duct irrigation was smooth at the one-week follow-up.

Several limitations of this study should be noted. First, this was a single-center cohort study without a contemporaneous control group; thus, the observed patency rate cannot definitively establish superiority over conventional irrigation or probing, and historical comparisons should be interpreted with caution. Second, as a pilot study, a formal *a priori* sample size calculation was not conducted prior to enrollment; nevertheless, *post hoc* estimation demonstrated that the achieved sample size of 166 eyes provided sufficient statistical precision (95% CI for overall patency: 77.4%–88.6%; 95% CI for complication rate: 1.3%–6.9%), laying a solid foundation for future multi-center randomized controlled trials. Third, age cohorts were observationally defined rather than randomized. Older infants (9–11 months) may represent a selected subgroup with longer disease duration, prior treatment failures, or structurally complex obstructions (selection/survivorship bias). Thus, the lower patency rate in older infants cannot be solely attributed to age, and findings regarding intervention timing should be interpreted as observational trends rather than definitive optimal windows. In addition, to preserve full clinical data integrity, all affected eyes from both unilateral and bilateral cases (*n* = 21, 14.5%) were included in the evaluation. Although each nasolacrimal duct represents an anatomically independent obstruction, the within-subject correlation in bilateral cases was not explicitly adjusted for in the statistical analysis, which represents a methodological limitation.

Given the safety and effectiveness of pressurized lacrimal duct irrigation demonstrated in this study, this technique may be integrated into the stepwise management of CNLDO. When conservative treatment and conventional lacrimal irrigation prove ineffective, the pressurized lacrimal duct irrigation technique described herein may be preferentially considered; if it also fails, other advanced surgical interventions may then be subsequently considered, such as lacrimal probing, lacrimal intubation, and DCR (30–32). Despite the promising efficacy of this modified technique, several clinical challenges remain, including long-term inflammation-induced mucosal thickening, structural lacrimal scarring, and the risk of procedural complications such as false tracts. Furthermore, for infants with complex nasolacrimal duct obstruction or those who have undergone multiple failed lacrimal duct irrigation and probing procedures, DCR serves as the last resort for CNLDO and is additionally indicated for children with bony obstruction or concurrent developmental anomalies that render other procedures unfeasible (33). Previous studies have shown that endoscopic dacryocystorhinostomy (endoDCR) is an effective and generally safe surgical option for pediatric nasolacrimal duct obstruction, with a high pooled patency rate and acceptable variability (34, 35). Given the immature skeletal development, limited surgical working space, and considerable difficulty of both surgery and postoperative follow-up in infants, endoDCR is recommended after 5 years of age (35–37). However, it may be performed earlier in cases with severe epiphora and purulent discharge that substantially impair the child's quality of life or in cases of secondary acute dacryocystitis (26).

To address these difficulties, establishing an intelligent, data-driven disease management framework for neonatal dacryocystitis is essential. Given the critical importance of identifying the optimal intervention timing, leveraging artificial intelligence (AI) to manage and evaluate longitudinal clinical datasets could assist in determining precise therapeutic windows and improving treatment outcomes. Indeed, AI-assisted management and risk prediction systems have already demonstrated substantial clinical benefits across various ophthalmic diseases (38–40). Integrating similar AI-guided management strategies into pediatric lacrimal care may offer a viable pathway to optimize individualized intervention strategies, reduce complications, and enhance overall procedural success.

## 5 Conclusions

In conclusion, the improved pressurized lacrimal duct irrigation technique with a modified needle is safe and effective for neonatal dacryocystitis as early as 3 months of age, with higher primary success observed in younger infants. For older infants, increasing the frequency of irrigation can also improve the patency rate of treatment.

## Data Availability

The original contributions presented in the study are included in the article/Supplementary Material, further inquiries can be directed to the corresponding authors.
